# Transactions of the Medical Society of the State of New York

**Published:** 1860-07

**Authors:** 


### BOOK AND PAMPHLET NOTICES.

---

Transactions of the Medical Society of the state of New York, for 1860.

The volume before us, kindly furnished by Secretary Dr. S. D. Willard, is moderate in size, but contains in concise form much that is valuable to the medical profession. The President, Dr. B. F. Barker, in his inaugural, alludes with just pride to the number and importance of the contributions to medical science which have come through this organization during the 62 years of its existence, claiming for it the paternity of the United States Pharmacopœia, and of the American Medical Association.

The report of the Committee on City Milk, by S. R. Percy, M. D., N. Y., and communicated by the New York Academy of Medicine, is exceedingly valuable, exhibiting the results of much laborious and accurate investigation upon the Pathology of " Swill Milk," and its effects upon children. The microscopic delineations are very fine indeed. The Board of Health of New York are much indebted to Dr. Willard for his able response to their request.

From the pen of Thomas W. Blatchford, A. M., M. D., we have " a condensed statement of what has been attempted for the advancement of Medical Education by the Medical Conventions of 1846 and 1847, and by the American Medical Association since its organization in 1847."

In the brief space of twelve pages, Dr. Blatchford presents before us the action and points of interest connected with this subject, as they appear running through the entire volume of the Transactions. An appropriate tribute is paid to the memory of deceased members of the Society, among whom we note the names, Silas West, M. D., of Binghamton; Jotham Purdy, M. D.; Joel E. Hawley, M. D., Levi Farr, M. D., and Frederick F. Backus, M. D.

The Statistical Tables and Mortuary Records are valuable for reference, and the members of the State, and officers of the County Societies are found in full.

Several very valuable contributions to Medicine and Surgery help to make it a volume of intrinsic worth, and a credit to the able body which it represents.

